# Fixed-Dose Combinations of Renin–Angiotensin System Inhibitors and Calcium Channel Blockers in the Treatment of Hypertension: A Comparison of Angiotensin Receptor Blockers and Angiotensin-Converting Enzyme Inhibitors

**DOI:** 10.1097/MD.0000000000002355

**Published:** 2015-12-28

**Authors:** Fu-Chih Hsiao, Ying-Chang Tung, Shing-Hsien Chou, Lung-Sheng Wu, Chia-Pin Lin, Chun-Li Wang, Yu-Sheng Lin, Chee-Jen Chang, Pao-Hsien Chu

**Affiliations:** From the Department of Cardiology, Chang Gung Memorial Hospital, Chang Gung University College of Medicine (F-CH, Y-CT, S-HC, L-SW, C-PL, C-LW, Y-SL, P-HC); Clinical Informatics and Medical Statistics Research Center, College of Medicine, Chang Gung University (C-J C); Healthcare Center, Chang Gung Memorial Hospital, Chang Gung University College of Medicine (Y-SL, P-HC); and Heart Failure Center, Chang Gung Memorial Hospital, Chang Gung University College of Medicine, Taipei, Taiwan (P-HC).

## Abstract

Fixed-dose combinations (FDCs) of different regimens are recommended in guidelines for the treatment of hypertension. However, clinical studies comparing FDCs of angiotensin receptor blocker (ARB)/calcium channel blocker (CCB) and angiotensin-converting enzyme inhibitor (ACE inhibitor)/CCB in hypertensive patients are lacking.

Using a propensity score matching of 4:1 ratio, this retrospective claims database study compared 2 FDC regimens, ARB/CCB and ACE inhibitor/CCB, in treating hypertensive patients with no known atherosclerotic cardiovascular disease. All patients were followed for at least 3 years or until the development of major adverse cardiovascular events (MACEs) during the study period. In addition, the effect of medication adherence on clinical outcomes was evaluated in subgroup analysis based on different portions of days covered.

There was no significant difference in MACE-free survival (hazard ratio [HR]: 1.21; 95% confidence interval [CI]: 0.98–1.50; *P* = 0.08) and survival free from hospitalization for heart failure (HR: 1.15; 95% CI: 082–1.61; *P* = 0.431), new diagnosis of chronic kidney disease (HR: 0.98; 95% CI: 071–1.36; *P* = 0.906), and initiation of dialysis (HR: 0.99; 95% CI: 050–1.92; *P* = 0.965) between the 2 study groups. The results remained the same within each subgroup of patients with different adherence statuses.

ARBs in FDC regimens with CCBs in the present study were shown to be as effective as ACE inhibitors at reducing the risks of MACEs, hospitalization for heart failure, new diagnosis of chronic kidney disease, and new initiation of dialysis in hypertensive patients, regardless of the medication adherence status.

## INTRODUCTION

Hypertension is the leading remediable risk factor for cardiovascular diseases, which are resulting in an estimated 9.4 million deaths worldwide annually.^1^ Clinical trials have shown that treatment for hypertension substantially reduces the incidence of cardiovascular outcomes, such as fatal and nonfatal myocardial infarction and stroke.^2,3^ Despite recent advances in medical therapy, hypertension is adequately controlled in only ∼13% of the diseased people worldwide,^4^ specifically 46.5% in the United States,^5^ and 24.5% in Taiwan,^6^ leading to the incentives to explore more effective hypertension treatment regimens.

A cornerstone of evidence-based hypertension treatment is the current guidelines of using renin–angiotensin system (RAS) inhibitors, including angiotensin-converting enzyme inhibitors (ACE inhibitors) and angiotensin receptor blockers (ARBs).^7–10^ For the majority of hypertensive patients, 2 or more antihypertensives are needed to achieve desirable blood pressures.^11^ The combination therapy of an RAS inhibitor with a calcium channel blocker (CCB) has been recommended.^7–10^

Nonadherence to a poly-pill regimen has been recognized as one of the main reasons for inadequate blood pressure control.^12^ Evidence suggests that a single-pill fixed-dose combination (FDC) more effectively controls blood pressure when compared with a free-equivalent combination or a monotherapy.^13^ The better medication compliance with FDC regimens may significantly reduce major adverse cardiac events (MACEs) and health care costs.^14^

The ACCOMPLISH study has demonstrated that FDCs of ACE inhibitor/CCB were superior at reducing mortality and cardiovascular events in hypertension management when compared with FDCs of ACE inhibitor/thiazide.^15^ However, the outcome data of FDCs of ARB/CCB in hypertension management are still lacking, even though ARBs are increasingly prescribed due to their less adverse effects than ACE inhibitors’, especially in Asian populations.^16–18^

As a result, there is a need to evaluate the cardiovascular outcomes of FDCs of ARB/CCB in hypertension treatment. Given the lack of large-scale randomized controlled trials, we designed a retrospective claims database analysis to compare the clinical outcomes of FDCs of ARB/CCB versus those of ACE inhibitor/CCB in real-world hypertension treatment.

## METHODS

The data included in this study were obtained from the National Health Insurance Research Database (NHIRD) of Taiwan. The National Health Insurance (NHI) program, a state-operated, universal health insurance program implemented since 1995, covers ∼99% of the entire Taiwanese population.^19–25^ The NHIRD contains inpatient registries from all medical facilities contracted with the National Health Insurance Administration and provides patient information including new-onset MACEs, which are classified with 1 principal and 4 secondary International Classification of Diseases, 9th Revision, Clinical Modification (ICD-9-CM) diagnosis codes. The Bureau of NHI encrypted all personal identifiers before information was released to researchers. Confidentiality was addressed by following the data processing regulations set by the Bureau of NHI. The Institutional Review Board approval was waived.

### Study Cohorts

**FIGURE 1:**
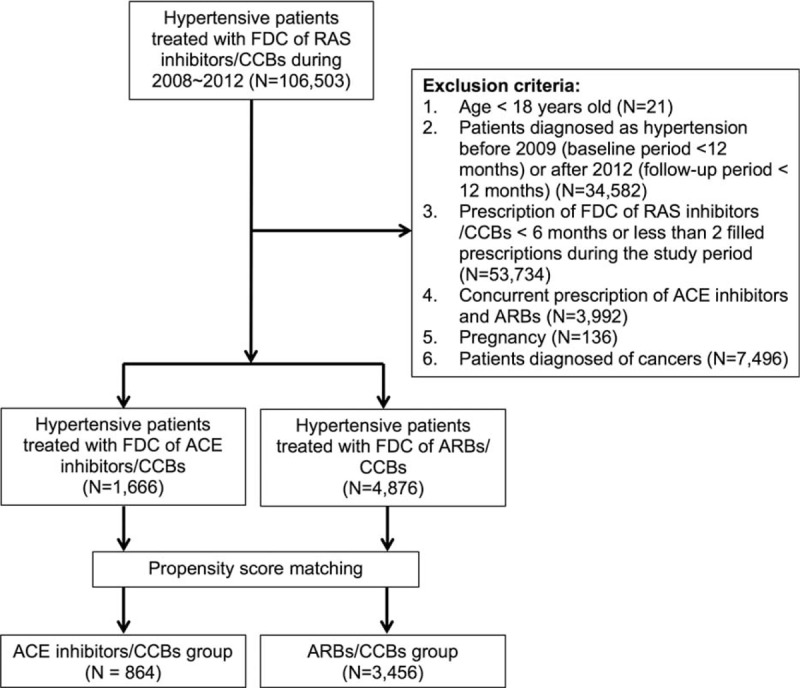
Patient enrollment.

Figure 1 shows the patient enrollment for this study. Two study cohorts of patients diagnosed with hypertension (ICD-9-CM: 401.x) from January of 2008 to December of 2012 were generated from the NHIRD. The first group consisted of those receiving FDCs of ACE inhibitor/CCB, and the second group consisted of those receiving FDCs of ARB/CCB. The date of first prescription of the studied medication was defined as the index date, and a period of 12 months preceding the index date was defined as the baseline period. Hypertensive patients who received any FDCs of RAS inhibitor/CCB during the baseline period were excluded from the study. To estimate the frequency of new-onset MACEs in a hypertensive population without established cardiovascular diseases, we also excluded hypertensive patients with previous diagnosis of coronary artery disease, myocardial infarction, stroke, peripheral artery disease, or heart failure before and during the baseline period. Other exclusion criteria were ages under 18, prescription duration of FDCs of RAS inhibitor/CCB for <6 months, concurrent prescription of ARBs and ACE inhibitors in the study period, pregnancy, and diagnosis of cancer.

We performed propensity score matching to avoid selection biases resulting from nonrandom assignment in this retrospective study. The variables used in the matching process were age, gender, dyslipidemia (ICD-9-CM: 272), diabetes mellitus (ICD-9-CM: 250), obesity (ICD-9-CM: 278), and chronic kidney disease (ICD-9-CM: 585). The ARB/CCB group was matched at a 4:1 ratio to the ACE inhibitor/CCB group.

The primary endpoints were defined as MACEs, including all-cause mortality, myocardial infarction (ICD-9-CM: 410–410.9), stroke (ICD-9-CM: 430–437), percutaneous coronary intervention (ICD-9-CM: 36.0–36.03 and 36.05–36.09), and coronary artery bypass surgery (ICD-9-CM: 36.1–36.99 and V45.81). Mortality was identified using death certificate data files. The secondary endpoints included hospitalization for heart failure, new diagnosis of chronic kidney disease, and initiation of dialysis also based on the endpoint morbidity-driven ICD-9-CM coding.

To evaluate the effect of patient adherence, we used the proportion of days covered (PDC) according to the insurance claims for the medications.^14,26^ Subgroup analysis was performed based on the status of medication adherence, that is PDC < 50%, PDC 50% to 80%, and PDC >80%. All patients were followed until the development of MACEs or for at least 3 years if no events occurred during the study period.

## STATISTICS

Continuous variables were compared using Student's *t* test, and categorical variables were analyzed by the chi square test. Data are presented as means, standard deviations, medians, or percentages. A logistic regression model was used for binary outcomes, and a Cox proportional hazard model was used for time to event analysis. All analyses were conducted using SAS Statistical Software, Version 9.3 (SAS Institute Inc, Cary, NC) and R Statistical Software, Version 3.0.1 (the R Foundation for Statistical Computing). A *P* value <0.05 was considered to be statistically significant.

## RESULTS

After propensity score matching, a total of 3456 patients receiving FDCs of ARB/CCB and 864 patients receiving FDCs of ACE inhibitor/CCB were enrolled. Table 1 demonstrates the demographic and baseline characteristics of the 2 groups. There were no significant differences between the 2 groups in terms of age and gender. Comorbidity conditions, including Charlson Comorbidity Score and number of cases of diabetes, chronic kidney disease, and dyslipidemia, were also statistically the same. Baseline medications and overall pill burden were similar between the 2 groups.

**TABLE 1:**
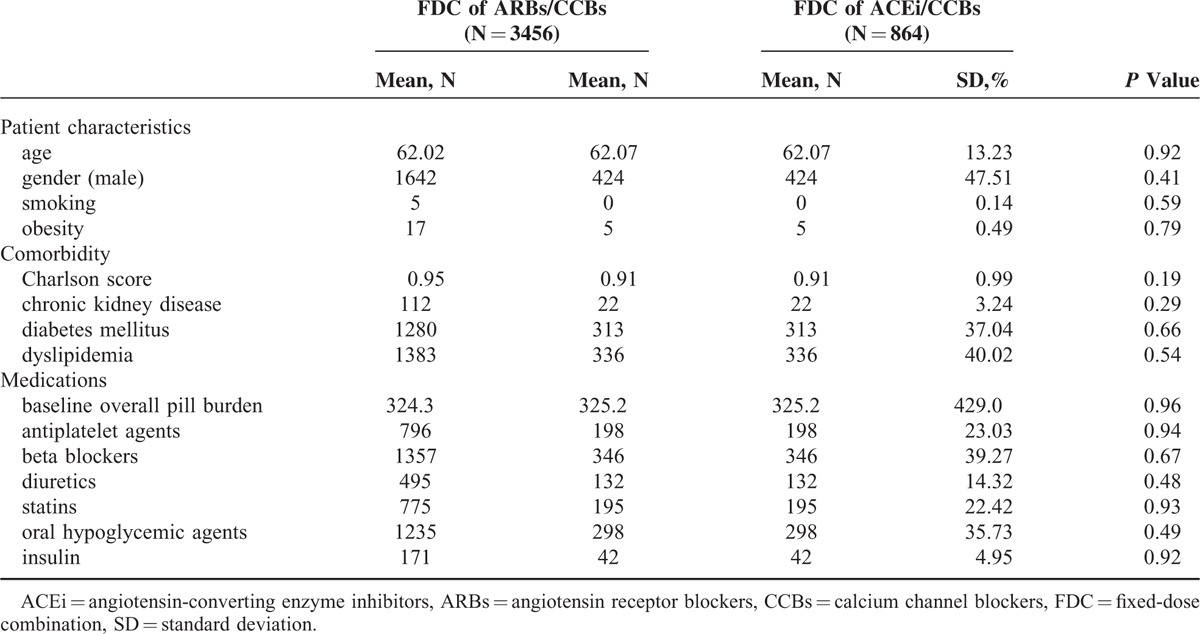
Baseline Characteristics of the Study Patients

**FIGURE 2:**
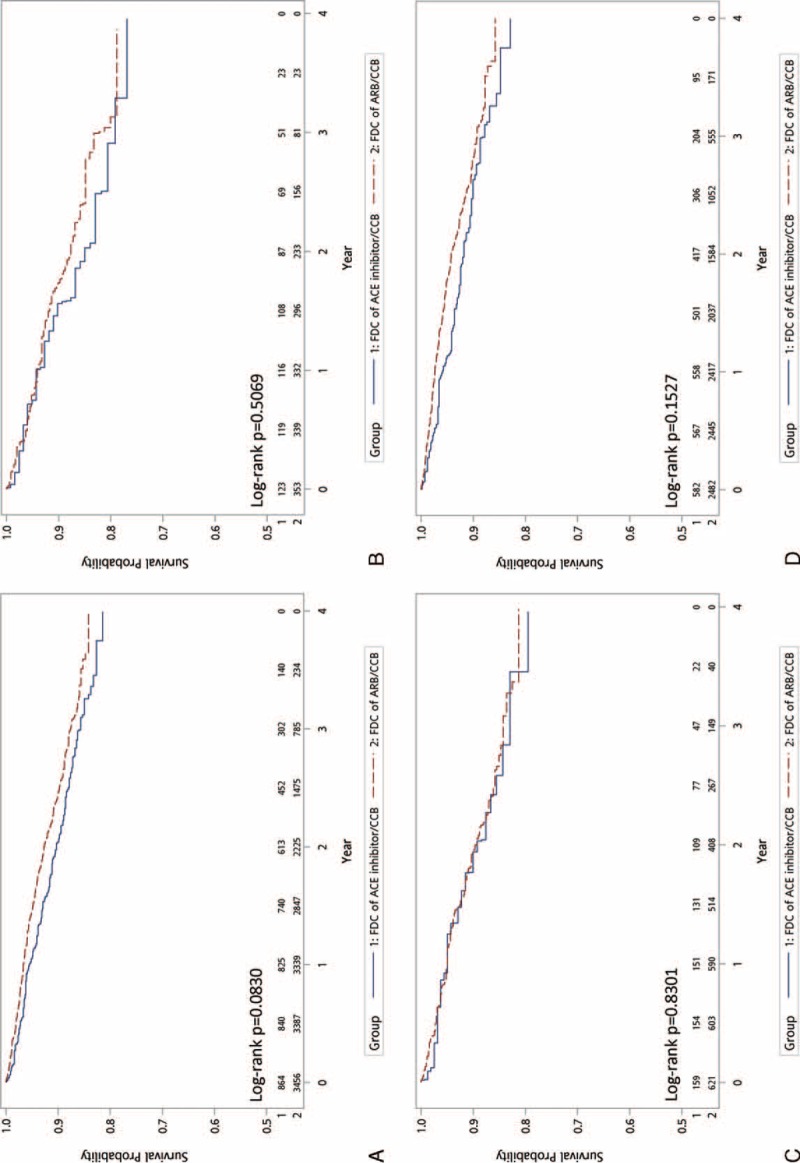
Comparison of the primary endpoints of FDCs of ARB/CCB versus ACE inhibitor/CCB: (A) all patient; (B) PDC < 50; (C) PDC = 50–80; (D) PDC ≥80. ACE = angiotensin-converting enzyme, ARB = angiotensin receptor blockers, CCB = calcium channel blockers, FDC = fixed-dose combination, PDC = proportion of days covered.

**FIGURE 3:**
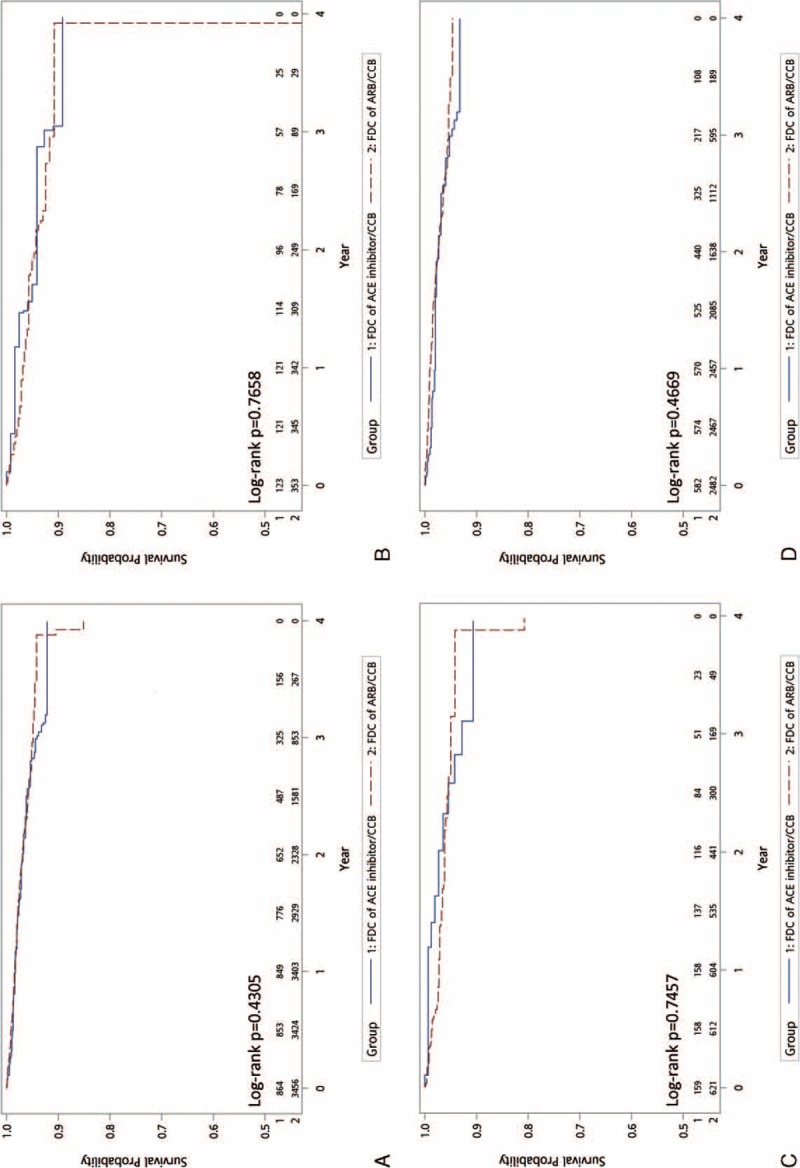
Comparison of the secondary endpoints of FDCs of ARB/CCB versus ACE inhibitor/CCB: hospitalization for heart failure—(A) all patients; (B) PDC < 50%; (C) PDC 50% to 80%; (D) PDC ≥80. ACE = angiotensin-converting enzyme, ARB = angiotensin receptor blockers, CCB = calcium channel blockers, FDC = fixed-dose combination, PDC = proportion of days covered.

**FIGURE 4:**
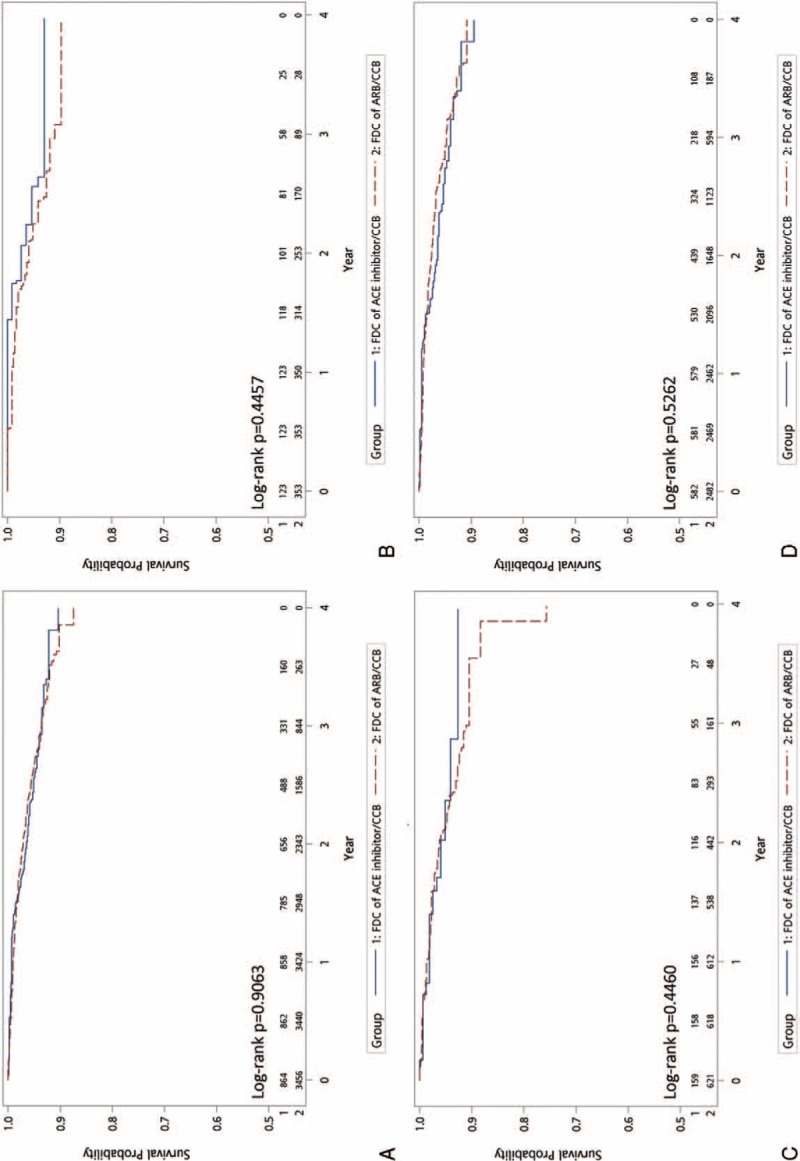
Comparison of the secondary endpoints of FDCs of ARB/CCB versus ACE inhibitor/CCB: new diagnosis of chronic kidney disease—(A) all patients; (B) PDC < 50%; (C) PDC 50% to 80%; (D) PDC ≥80. ACE = angiotensin-converting enzyme, ARB = angiotensin receptor blockers, CCB = calcium channel blockers, FDC = fixed-dose combination, PDC = proportion of days covered.

**FIGURE 5:**
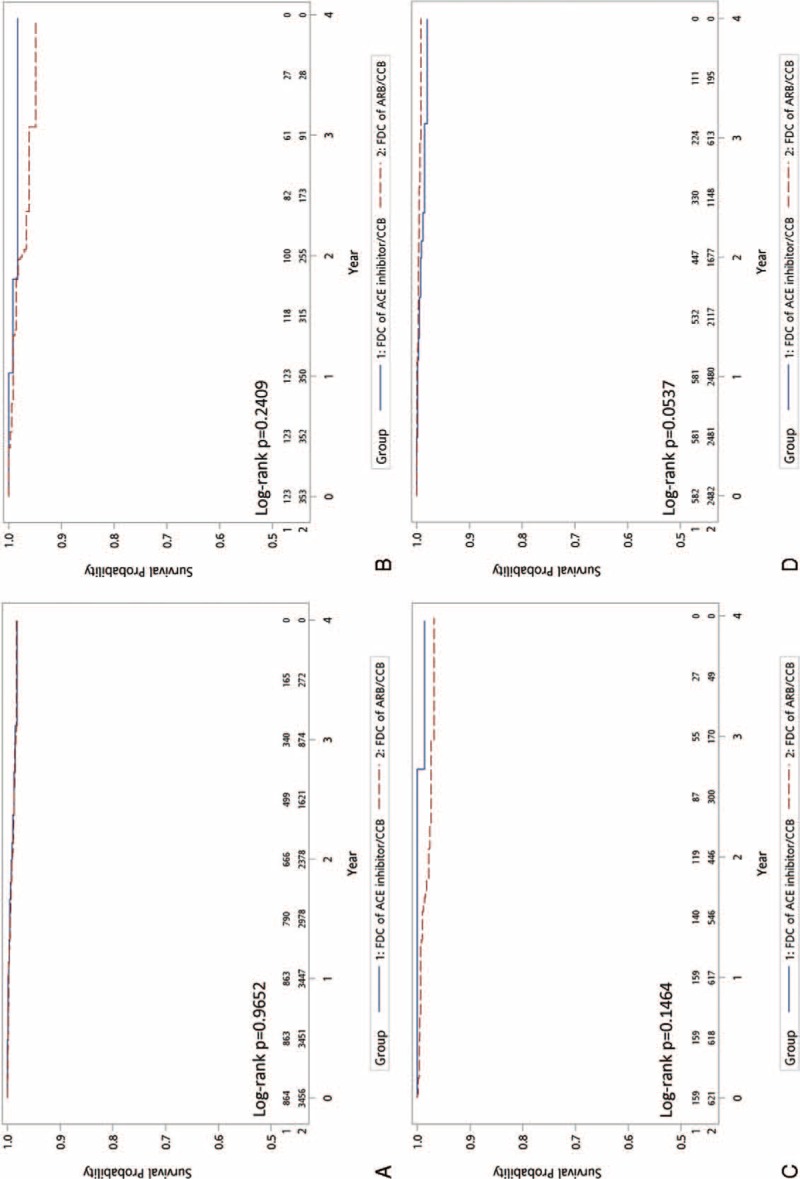
Comparison of the secondary endpoints of FDCs of ARB/CCB versus ACE inhibitor/CCB: initiation of dialysis—(A) all patients; (B) PDC < 50%; (C) PDC 50% to 80%; (D) PDC ≥80. ACE = angiotensin-converting enzyme, ARB = angiotensin receptor blockers, CCB = calcium channel blockers, FDC = fixed-dose combination, PDC = proportion of days covered.

Figure 2 shows the Kaplan–Meier curves of MACE-free survival and demonstrates no significant difference between the 2 groups (HR: 1.21; 95% CI: 0.98–1.50; *P* = 0.083). In terms of secondary outcomes, including hospitalization for heart failure (Fig. 3A, HR: 1.15; 95% CI: 0.82–1.61; *P* = 0.431), new diagnosis of chronic kidney disease (Fig. 4A, HR: 0.98; 95% CI: 0.71–1.36; *P* = 0.906), and initiation of dialysis (Fig. 5A, HR: 0.99; 95% CI: 0.50–1.92; *P* = 0.965), no significant difference between the 2 treatment groups was observed.

We divided the patients into 3 categories according to the medication adherence status for subgroup analysis. Figures 3B–D, 4B–D, and 5B–D demonstrate that, regardless of the PDC, both primary and secondary outcomes were comparable for FDCs of ARB/CCB and ACE inhibitor/CCB.

## DISCUSSION

This retrospective claims database analysis compared clinical outcomes of 2 FDC regimens, ARB/CCB and ACE inhibitor/CCB, for hypertensive patients with no established cardiovascular diseases. All patients were followed for at least 3 years or until the development of MACEs. Overall, the FDCs of ARB/CCB had comparable primary and secondary outcomes to those of ACE inhibitor/CCB, regardless of the adherence status.

Inhibition of the RAS has become a major pharmaceutical biomedical objective in hypertension treatment as elevated RAS activity and high blood pressure are closely related. RAS inhibition has also been recognized as the cornerstone of evidence-based therapies for patients with high cardiovascular risk, left ventricular dysfunction after myocardial infarction, and heart failure.^27–29^

Evidence consistently demonstrates ACE inhibitors’ efficacy in reducing mortality and MACEs for hypertensive patients.^30^ However, a meta-analysis conducted by Roberto Ferrari et al reported that the effect of treatment with ACE inhibitors on all-cause mortality was significant but that of treatment with ARBs was not.^30^ In diabetic patients, another recent meta-analysis demonstrated that ACE inhibitors reduced all-cause mortality, cardiovascular mortality, and MACEs, whereas ARBs did not.^31^ Strippoli et al's meta-analysis also showed that ACE inhibitors, but not ARBs, reduced all-cause mortality in patients with diabetic nephropathy.^32^ On the contrary, the ONTARGET trial, the largest randomized trial, reported equal potency of telmisartan, an ARB, and ramipril, an ACE inhibitor, at reducing cardiovascular events and death in patients with cardiovascular disease or high-risk diabetes.^33^ Similarly, the VALIANT trial showed that valsartan, another ARB, was as effective as ACE inhibitors at reducing cardiovascular morbidity and mortality in patients with left ventricular dysfunction after acute myocardial infarction.^34^ An increasing number of clinical trials have demonstrated that ARBs are just as effective as ACE inhibitors at blood pressure reduction,^35^ heart failure symptoms improvement,^36^ diabetic nephropathy prevention,^37,38^ stroke reduction,^39^ and type 2 diabetes mellitus reduction.^40^

Despite previous controversial results of the effectiveness of ARBs and ACE inhibitors, more recent meta-analysis studies concluded that the available evidence did not support a difference in overall mortality or cardiovascular outcomes between ARBs and ACE inhibitors.^41–43^ What is more is that ARBs’ intra-class differences in pharmacodynamic and pharmacokinetic properties may also contribute to the inconsistency in therapeutic effects as well as clinical outcomes beyond blood pressure control.^44^ In addition, the functional role of adrenergic system in hypertension and its complications are also related to the cardiovascular health in elderly patients.^45,46^ Lymperopoulos et al elucidated that through the suppression of the β-arrestin 1-dependent signaling pathway, candesartan and valsartan are more potent than other ARBs at blocking adrenal aldosterone synthesis, which may translate to their superior clinical benefits in attenuating postmyocardial infarction remodeling and progression to heart failure.^47,48^

For the majority of hypertensive patients, 2 or more antihypertensive agents are needed to achieve target blood pressure values.^11^ Combinations of 2 antihypertensive agents in a single pill have been shown to improve medication compliance^49–52^ and have therefore been recommended by hypertension guidelines.^7–10^ At least 30 clinical trials have compared different combination regimens with a placebo, a monotherapy, or other combinations.^8^ The blood pressure-lowering arm of the ASCOT-BPLA study was among the first studies to document the efficacy of a combination of an RAS inhibitor and a CCB in hypertension management.^53^ Furthermore, the ACCOMPLISH trial, which was a randomized, double-blind trial assigning 11,506 hypertensive patients at high risk of cardiovascular events to receive treatment with either benazepril/amlodipine or benazepril/hydrochlorothiazide, demonstrated that an FDC of ACE inhibitor/CCB (benazepril/amlodipine) was superior to an FDC of ACE inhibitor/thiazide at reducing cardiovascular events.^15^

Several small trials have confirmed the combination effect of ARBs and CCBs at reducing blood pressure.^54–58^ However, no trial has ever been performed to compare FDCs of ARB/CCB head-to-head with those of ACE inhibitor/CCB based on cardiovascular event outcomes. Although large-scale, randomized controlled trials are still not available, we designed a nationwide retrospective claims database analysis to draw such comparisons. In the present study, we demonstrated that FDC regimens of ARB/CCB were comparable to that of ACE inhibitor/CCB at reducing MACEs, hospitalization for heart failure, new diagnosis of chronic kidney disease, and initiation of dialysis. Our findings were consistent with the previous studies in showing the beneficial results of combining ARBs and CCBs, and support the use of FDCs of ARB/CCB as a valuable alternative to those of ACE inhibitor/CCB in hypertension management.

When we divided the patients into 3 categories according to their medication adherence status for subgroup analysis, there were no significant differences in both primary and secondary outcomes between FDCs of ARB/CCB and FDCs of ACE inhibitor/CCB in each subgroup. A possible explanation is that all data used in this study came from prescription information provided by individual physicians, which was not originally intended for study purposes. In a database analysis, it would be difficult to determine whether the prescribed medications were actually taken, and therefore, the use of PDC may over- or underestimate actual medication adherence.

As this retrospective cohort study was based on a claims database, there were inevitably other inherent limitations. For example, coding errors and typos are not uncommon in real-world practice. In addition, blood pressure, an important measurement of the efficacy of antihypertensive agents, could not be obtained at the baseline period or during follow-up periods. Possible risk factors such as smoking and socioeconomic status, and their effects were also difficult to be well assessed in this study.

Furthermore, the study group of the present analysis is limited to the Taiwanese population, which may not be representative of the Asian population or the world population. Additionally, there is growing evidence suggesting that single nucleotide polymorphisms of different genes, such as Pl(A1/A2), CaMK4 and G-protein-coupled receptor kinase 2, among different ethnic groups may affect the incidence of hypertension and the cardiovascular complications of hypertension.^59–62^ Finally, due to limitation of the number of patients studied, we did not perform further analyses regarding any association between intra-class differences of RAS inhibitors and clinical outcomes; neither did we perform subgroup analyses based on comorbidities such as diabetes and chronic kidney disease. A prospective randomized controlled trial is warranted for further validation of our results.

## CONCLUSION

In this retrospective database study in Taiwan, ARBs when compared with ACE inhibitors in FDC regimens that include CCBs were shown to be comparably effective at reducing the risks of MACEs, hospitalization for heart failure, new diagnosis of chronic kidney disease, and new initiation of dialysis in hypertensive patients with no established cardiovascular diseases. The results remained the same for patients across all adherence statuses.

## References

[1] Angell SY De Cock KM Frieden TR A public health approach to global management of hypertension. *Lancet* 2015; 385:825–827. 25752181 10.1016/S0140-6736(14)62256-X PMC4830267

[2] Kannel WB Blood pressure as a cardiovascular risk factor: prevention and treatment. *JAMA* 1996; 275:1571–1576. 8622248

[3] Mulrow CD Pignone M What are the elements of good treatment for hypertension? *BMJ* 2001; 322:1107–1109. 11337444 10.1136/bmj.322.7294.1107 PMC1120238

[4] Chow CK Teo KK Rangarajan S Prevalence, awareness, treatment, and control of hypertension in rural and urban communities in high-, middle-, and low-income countries. *JAMA* 2013; 310:959–968. 24002282 10.1001/jama.2013.184182

[5] Guo F He D Zhang W Trends in prevalence, awareness, management, and control of hypertension among United States adults, 1999 to 2010. *J Am Coll Cardiol* 2012; 60:599–606. 22796254 10.1016/j.jacc.2012.04.026

[6] Su TC Bai CH Chang HY Evidence for improved control of hypertension in Taiwan: 1993–2002. *J Hypertens* 2008; 26:600–606. 18300873 10.1097/HJH.0b013e3282f3b352

[7] James PA Oparil S Carter BL 2014 evidence-based guideline for the management of high blood pressure in adults: report from the panel members appointed to the Eighth Joint National Committee (JNC 8). *JAMA* 2014; 311:507–520. 24352797 10.1001/jama.2013.284427

[8] Mancia G Fagard R Narkiewicz K 2013 ESH/ESC Practice Guidelines for the Management of Arterial Hypertension. *Blood Press* 2014; 23:3–16. 24359485 10.3109/08037051.2014.868629

[9] Rosendorff C Lackland DT Allison M Treatment of hypertension in patients with coronary artery disease: a scientific statement from the American Heart Association, American College of Cardiology, and American Society of Hypertension. *J Am Coll Cardiol* 2015; 65:1998–2038. 25840655 10.1016/j.jacc.2015.02.038

[10] Chiang CE Wang TD Ueng KC 2015 guidelines of the Taiwan Society of Cardiology and the Taiwan Hypertension Society for the management of hypertension. *J Chin Med Assoc* 2015; 78:1–47. 25547819 10.1016/j.jcma.2014.11.005

[11] Gradman AH Basile JN Carter BL Combination therapy in hypertension. *J Am Soc Hypertens* 2010; 4:90–98. 20400053 10.1016/j.jash.2010.03.001

[12] Elliott WJ Improving outcomes in hypertensive patients: focus on adherence and persistence with antihypertensive therapy. *J Clin Hypertens (Greenwich)* 2009; 11:376–382. 19583634 10.1111/j.1751-7176.2009.00138.x PMC8673138

[13] Egan BM Bandyopadhyay D Shaftman SR Initial monotherapy and combination therapy and hypertension control the first year. *Hypertension* 2012; 59:1124–1131. 22566499 10.1161/HYPERTENSIONAHA.112.194167 PMC3425944

[14] Tung YC Lin YS Wu LS Clinical outcomes and healthcare costs in hypertensive patients treated with a fixed-dose combination of amlodipine/valsartan. *J Clin Hypertens (Greenwich)* 2015; 17:51–58. 25477188 10.1111/jch.12449 PMC8031572

[15] Jamerson K Weber MA Bakris GL Benazepril plus amlodipine or hydrochlorothiazide for hypertension in high-risk patients. *N Engl J Med* 2008; 359:2417–2428. 19052124 10.1056/NEJMoa0806182

[16] Woo KS Nicholls MG High prevalence of persistent cough with angiotensin converting enzyme inhibitors in Chinese. *Br J Clin Pharmacol* 1995; 40:141–144. 8562296 PMC1365173

[17] Ng LP Goh PS Incidence of discontinuation of angiotensin-converting enzyme inhibitors due to cough, in a primary healthcare centre in Singapore. *Singapore Med J* 2014; 55:146–149. 24664381 10.11622/smedj.2014034 PMC4293986

[18] Hsu CN Wang TD Secular trends in prescription patterns of single-pill combinations of an angiotensin-converting enzyme inhibitor or angiotensin receptor blocker plus a thiazide diuretic for hypertensive patients in Taiwan. *Acta Cardiol Sin* 2013; 29:49–55. PMC4804960 27122684

[19] Boyko EJ Barr EL Zimmet PZ Two-hour glucose predicts the development of hypertension over 5 years: the AusDiab study. *J Hum Hypertens* 2008; 22:168–176. 18046430 10.1038/sj.jhh.1002316

[20] Chiang CH Huang WC Yang JS Five-year outcomes after acute myocardial infarction in patients with and without diabetes mellitus in Taiwan, 1996–2005. *Acta Cardiol Sin* 2013; 29:387–394. PMC4804787 27122735

[21] Chou SH Tung YC Lin YS Major adverse cardiovascular events in treated periodontitis: a population-based follow-up study from Taiwan. *PloS One* 2015; 10:e0130807. 26114433 10.1371/journal.pone.0130807 PMC4482590

[22] Lin YS Liu PH Wu LS Major adverse cardiovascular events in adult congenital heart disease: a population-based follow-up study from Taiwan. *BMC Cardiovasc Disord* 2014; 14:38. 24655794 10.1186/1471-2261-14-38 PMC3994523

[23] Lin YS Tang CH Yang CY Effect of pre-eclampsia-eclampsia on major cardiovascular events among peripartum women in Taiwan. *Am J Cardiol* 2011; 107:325–330. 21211611 10.1016/j.amjcard.2010.08.073

[24] Tang CH Wu CS Lee TH Preeclampsia-eclampsia and the risk of stroke among peripartum in Taiwan. *Stroke* 2009; 40:1162–1168. 19228854 10.1161/STROKEAHA.108.540880

[25] Wu LS Tang CH Lin YS Major adverse cardiovascular events and mortality in systemic lupus erythematosus patients after successful delivery: a population-based study. *Am J Med Sci* 2014; 347:42–49. 23255242 10.1097/MAJ.0b013e318278707f

[26] Cramer JA Roy A Burrell A Medication compliance and persistence: terminology and definitions. *Value Health* 2008; 11:44–47. 18237359 10.1111/j.1524-4733.2007.00213.x

[27] Rosendorff C Black HR Cannon CP Treatment of hypertension in the prevention and management of ischemic heart disease: a scientific statement from the American Heart Association Council for High Blood Pressure Research and the Councils on Clinical Cardiology and Epidemiology and Prevention. *Circulation* 2007; 115:2761–2788. 17502569 10.1161/CIRCULATIONAHA.107.183885

[28] O’Gara PT Kushner FG Ascheim DD 2013 ACCF/AHA guideline for the management of ST-elevation myocardial infarction: a report of the American College of Cardiology Foundation/American Heart Association Task Force on Practice Guidelines. *Circulation* 2013; 127:e362–425. 23247304 10.1161/CIR.0b013e3182742cf6

[29] Yancy CW Jessup M Bozkurt B 2013 ACCF/AHA guideline for the management of heart failure: executive summary: a report of the American College of Cardiology Foundation/American Heart Association Task Force on practice guidelines. *Circulation* 2013; 128:1810–1852. 23741057 10.1161/CIR.0b013e31829e8807

[30] Ferrari R Boersma E The impact of ACE inhibition on all-cause and cardiovascular mortality in contemporary hypertension trials: a review. *Expert Rev Cardiovasc Ther* 2013; 11:705–717. 23750680 10.1586/erc.13.42

[31] Cheng J Zhang W Zhang X Effect of angiotensin-converting enzyme inhibitors and angiotensin II receptor blockers on all-cause mortality, cardiovascular deaths, and cardiovascular events in patients with diabetes mellitus: a meta-analysis. *JAMA Intern Med* 2014; 174:773–785. 24687000 10.1001/jamainternmed.2014.348

[32] Strippoli GF Craig M Deeks JJ Effects of angiotensin converting enzyme inhibitors and angiotensin II receptor antagonists on mortality and renal outcomes in diabetic nephropathy: systematic review. *BMJ* 2004; 329:828. 15459003 10.1136/bmj.38237.585000.7C PMC521570

[33] Investigators O Yusuf S Teo KK Telmisartan, ramipril, or both in patients at high risk for vascular events. *N Engl J Med* 2008; 358:1547–1559. 18378520 10.1056/NEJMoa0801317

[34] McMurray J Solomon S Pieper K The effect of valsartan, captopril, or both on atherosclerotic events after acute myocardial infarction: an analysis of the Valsartan in Acute Myocardial Infarction Trial (VALIANT). *J Am Coll Cardiol* 2006; 47:726–733. 16487836 10.1016/j.jacc.2005.09.055

[35] Probstfield JL O’Brien KD Progression of cardiovascular damage: the role of renin-angiotensin system blockade. *Am J Cardiol* 2010; 105 (1 Suppl):10A–20A. 20102969 10.1016/j.amjcard.2009.10.006

[36] Lee VC Rhew DC Dylan M Meta-analysis: angiotensin-receptor blockers in chronic heart failure and high-risk acute myocardial infarction. *Ann Intern Med* 2004; 141:693–704. 15520426 10.7326/0003-4819-141-9-200411020-00011

[37] Andersen S Tarnow L Rossing P Renoprotective effects of angiotensin II receptor blockade in type 1 diabetic patients with diabetic nephropathy. *Kidney Int* 2000; 57:601–606. 10652037 10.1046/j.1523-1755.2000.00880.x

[38] Lacourciere Y Belanger A Godin C Long-term comparison of losartan and enalapril on kidney function in hypertensive type 2 diabetics with early nephropathy. *Kidney Int* 2000; 58:762–769. 10916100 10.1046/j.1523-1755.2000.00224.x

[39] Turnbull F Neal B Blood Pressure Lowering Treatment Trialists Collaboration. Blood pressure-dependent and independent effects of agents that inhibit the renin-angiotensin system. *J Hypertens* 2007; 25:951–958. 17414657 10.1097/HJH.0b013e3280bad9b4

[40] Abuissa H Jones PG Marso SP Angiotensin-converting enzyme inhibitors or angiotensin receptor blockers for prevention of type 2 diabetes: a meta-analysis of randomized clinical trials. *J Am Coll Cardiol* 2005; 46:821–826. 16139131 10.1016/j.jacc.2005.05.051

[41] Matchar DB McCrory DC Orlando LA Systematic review: comparative effectiveness of angiotensin-converting enzyme inhibitors and angiotensin II receptor blockers for treating essential hypertension. *Ann Intern Med* 2008; 148:16–29. 17984484 10.7326/0003-4819-148-1-200801010-00189

[42] Li EC Heran BS Wright JM Angiotensin converting enzyme (ACE) inhibitors versus angiotensin receptor blockers for primary hypertension. *Cochrane Database Syst Rev* 2014; 8:CD009096. 25148386 10.1002/14651858.CD009096.pub2 PMC6486121

[43] Savarese G Costanzo P Cleland JG A meta-analysis reporting effects of angiotensin-converting enzyme inhibitors and angiotensin receptor blockers in patients without heart failure. *J Am Coll Cardiol* 2013; 61:131–142. 23219304 10.1016/j.jacc.2012.10.011

[44] Baumhakel M Bohm M Cardiovascular outcomes with angiotensin II receptor blockers: clinical implications of recent trials. *Vasc Health Risk Manag* 2011; 7:391–397. 21796253 10.2147/VHRM.S17168 PMC3141911

[45] Santulli G Adrenal signaling in heart failure something more than a distant ship's smoke on the horizon. *Hypertension* 2014; 63:215–216. 24218430 10.1161/HYPERTENSIONAHA.113.02382

[46] Santulli G Ciccarelli M Trimarco B Physical activity ameliorates cardiovascular health in elderly subjects: the functional role of the beta adrenergic system. *Front Physiol* 2013; 4:209. 23964243 10.3389/fphys.2013.00209 PMC3740240

[47] Lymperopoulos A Sturchler E Bathgate-Siryk A Different potencies of angiotensin receptor blockers at suppressing adrenal beta-Arrestin1-dependent post-myocardial infarction hyperaldosteronism. *J Am Coll Cardiol* 2014; 64:2805–2806. 25541135 10.1016/j.jacc.2014.09.070

[48] Dabul S Bathgate-Siryk A Valero TR Suppression of adrenal betaarrestin1-dependent aldosterone production by ARBs: head-to-head comparison. *Sci Rep* 2015; 5:8116. 25631300 10.1038/srep08116 PMC4309955

[49] Wan XMP Zhang X A promising choice in hypertension treatment: fixed-dose combinations. *Asian J Pharm Sci* 2014; 9:7.

[50] Dickson M Plauschinat CA Compliance with antihypertensive therapy in the elderly: a comparison of fixed-dose combination amlodipine/benazepril versus component-based free-combination therapy. *Am J Cardiovasc Drugs* 2008; 8:45–50. 18303937 10.2165/00129784-200808010-00006

[51] Sanz G Fuster V Fixed-dose combination therapy and secondary cardiovascular prevention: rationale, selection of drugs and target population. *Nat Clin Pract Cardiovasc Med* 2009; 6:101–110. 19104519 10.1038/ncpcardio1419

[52] van Galen KA Nellen JF Nieuwkerk PT The effect on treatment adherence of administering drugs as fixed-dose combinations versus as separate pills: systematic review and meta-analysis. *AIDS Res Treat* 2014; 2014:967073. 25276422 10.1155/2014/967073 PMC4168145

[53] Dahlof B Sever PS Poulter NR Prevention of cardiovascular events with an antihypertensive regimen of amlodipine adding perindopril as required versus atenolol adding bendroflumethiazide as required, in the Anglo-Scandinavian Cardiac Outcomes Trial-Blood Pressure Lowering Arm (ASCOT-BPLA): a multicentre randomised controlled trial. *Lancet* 2005; 366:895–906. 16154016 10.1016/S0140-6736(05)67185-1

[54] Santulli G Adrenal signaling in heart failure: something more than a distant ship's smoke on the horizon. *Hypertension* 2014; 63:215–216. 24218430 10.1161/HYPERTENSIONAHA.113.02382

[55] Santulli G Ciccarelli M Trimarco B Physical activity ameliorates cardiovascular health in elderly subjects: the functional role of the beta adrenergic system. *Front Physiol* 2013; 4:209. 23964243 10.3389/fphys.2013.00209 PMC3740240

[56] Yamaguchi J Ogawa H Hagiwara N Indication and advantage of combination therapy with angiotensin II receptor blocker (ARB) and calcium channel antagonist. *Nihon Rinsho* 2011; 69:2059–2063. 22111330

[57] Bobrie G Investigators IAS I-ADD study: assessment of efficacy and safety profile of irbesartan/amlodipine fixed-dose combination therapy compared with irbesartan monotherapy in hypertensive patients uncontrolled with irbesartan 150 mg monotherapy: a multicenter, phase III, prospective, randomized, open-label with blinded-end point evaluation study. *Clin Ther* 2012; 34:1720–1734.e1723. 22853847 10.1016/j.clinthera.2012.07.001

[58] Bobrie G Investigators ICS I-COMBINE study: assessment of efficacy and safety profile of irbesartan/amlodipine fixed-dose combination therapy compared with amlodipine monotherapy in hypertensive patients uncontrolled with amlodipine 5 mg monotherapy: a multicenter, phase III, prospective, randomized, open-label with blinded-end point evaluation study. *Clin Ther* 2012; 34:1705–1719. 22853848 10.1016/j.clinthera.2012.06.026

[59] Lanni F Santulli G Izzo R The Pl(A1/A2) polymorphism of glycoprotein IIIa and cerebrovascular events in hypertension: increased risk of ischemic stroke in high-risk patients. *J Hypertens* 2007; 25:551–556. 17278970 10.1097/HJH.0b013e328013cd67

[60] Galasso G Santulli G Piscione F The GPIIIA PlA2 polymorphism is associated with an increased risk of cardiovascular adverse events. *BMC Cardiovasc Disord* 2010; 10:41. 20846430 10.1186/1471-2261-10-41 PMC2954874

[61] Santulli G Cipolletta E Sorriento D CaMK4 gene deletion induces hypertension. *J Am Heart Assoc* 2012; 1:e001081. 23130158 10.1161/JAHA.112.001081 PMC3487344

[62] Santulli G Trimarco B Iaccarino G G-protein-coupled receptor kinase 2 and hypertension: molecular insights and pathophysiological mechanisms. *High Blood Press Cardiovasc Prev* 2013; 20:5–12. 23532739 10.1007/s40292-013-0001-8

